# Supplementary material for: Deep learning-based segmentation of 2D projection-derived overlapping prospore membrane in yeast
Source: Cell Struct Funct. 2025 Sep 13;50(2):183–95. doi: 10.1247/csf.25032 (PMC12967522; doi:10.1247/csf.25032)
Supplement: Supplementary file 3 — Supplementary Materials [file csf_50_25032_3.zip › 50_25032_Supplementary Table.docx]

**Supplementary table 1. Yeast strains and plasmids used in this study.**

Strain Name Genotype Source

TNY1218 *MAT***a**/*MAT*α *his3*Δ*SK*/*his3*Δ*SK ura3*/*ura3::pRS306-mCherry-Spo20^51-91^ trp1::hisG*/*trp1::hisG leu2*/*leu2 arg4-NspI/ARG4*

*lys2/lys2 ho*Δ*::LYS2/ho*Δ*::LYS2 rme1::LEU2/RME1*

*AUR1::P_ACT1_-LexA-ER-haVP16::AUR1-C/AUR1::P_ACT1_-LexA-ER-haVP16::AUR1-C*

*NDT80::hphNT1::P4×lexA-9×Myc-NDT80* /*NDT80::hphNT1::P4×lexA-9×Myc-NDT80* ^1^

YSIY793 *MAT***a**/*MAT*α *his3*Δ*SK*/*his3*Δ*SK ura3*/*ura3::pRS306-mCherry-Spo20^51-91^ trp1::hisG*/*trp1::hisG leu2*/*leu2 arg4-NspI/ARG4*

*lys2/lys2 ho*Δ*::LYS2/ho*Δ*::LYS2 rme1::LEU2/RME1*

*AUR1::P_ACT1_-LexA-ER-haVP16::AUR1-C/AUR1::P_ACT1_-LexA-ER-haVP16::AUR1-C*

*NDT80::hphNT1::P4×lexA-9×Myc-NDT80* /*NDT80::hphNT1::P4×lexA-9×Myc-NDT80* *gip1*∆*::kanMX6/gip1*∆*::kanMX6* ^1^

Plasmid Name Description Source

pRS306-mCherry-Spo20^51-91^ *URA3, integration, P_TEF1_-mCherry-Spo20^51-91^* ^1^

1. Suda, Y., Tachikawa, H., Suda, T., Kurokawa, K., Nakano, A., and Irie, K. (2024). Remodeling of the secretory pathway is coordinated with de novo membrane formation in budding yeast gametogenesis. iScience *27*, 110855. https://doi.org/10.1016/j.isci.2024.110855.
